# A global map of the protein shape universe

**DOI:** 10.1371/journal.pcbi.1006969

**Published:** 2019-04-12

**Authors:** Xusi Han, Atilla Sit, Charles Christoffer, Siyang Chen, Daisuke Kihara

**Affiliations:** 1 Department of Biological Sciences, Purdue University, West Lafayette, Indiana, United States of America; 2 Department of Mathematics & Statistics, Eastern Kentucky University, Richmond, Kentucky, United States of America; 3 Department of Computer Science, Purdue University, West Lafayette, Indiana, United States of America; Columbia University, UNITED STATES

## Abstract

Proteins are involved in almost all functions in a living cell, and functions of proteins are realized by their tertiary structures. Obtaining a global perspective of the variety and distribution of protein structures lays a foundation for our understanding of the building principle of protein structures. In light of the rapid accumulation of low-resolution structure data from electron tomography and cryo-electron microscopy, here we map and classify three-dimensional (3D) surface shapes of proteins into a similarity space. Surface shapes of proteins were represented with 3D Zernike descriptors, mathematical moment-based invariants, which have previously been demonstrated effective for biomolecular structure similarity search. In addition to single chains of proteins, we have also analyzed the shape space occupied by protein complexes. From the mapping, we have obtained various new insights into the relationship between shapes, main-chain folds, and complex formation. The unique view obtained from shape mapping opens up new ways to understand design principles, functions, and evolution of proteins.

## Author summary

Proteins are the major molecules involved in almost all cellular processes. In this work, we present a novel mapping of protein shapes that represents the variety and the similarities of 3D shapes of proteins and their assemblies. This mapping provides various novel insights into protein shapes including determinant factors of protein 3D shapes, which enhance our understanding of the design principles of protein shapes. The mapping will also be a valuable resource for artificial protein design as well as references for classifying medium- to low-resolution protein structure images of determined by cryo-electron microscopy and tomography.

## Introduction

Proteins are the primary workers in a living cell, involved in transportation, catalysis, signaling, energy production, and many other processes. Classification of protein structures provides fundamental information for our understanding of the principles that govern and determine protein structures, which is one of the essential goals of structural biology and protein bioinformatics. Understanding the repertoire of protein structures is also of practical importance for artificial protein design, which has broad applications in therapeutics such as designing inhibitors [1] and small peptide drugs [2], as well as the development of biomaterials [3].

Conventionally, protein structures have been classified based on their main-chain conformations and evolutionary history [4–6]. Such classifications led to several important observations including the number of different protein folds in nature [7–9], distributions of folds in genomes [10,11], and the relationship between sequence and structure conservations [12]. The discovery of the limited number of folds yielded stimulating discussions on the mechanism behind it [13,14]. Furthermore, such studies contributed to the birth of a very successful paradigm of threading [15] and more recent fragment-based approaches [16] in protein structure prediction.

Some recent studies mapped protein structures into a low-dimensional space to reveal high-level organization of the variety of protein structures. Kim and his colleagues computed structural similarity with DALI, a residue-contact map-based structure comparison method [17], and mapped representative proteins into a 3D space using multidimensional scaling [18,19]. Osadchy and Kolodny represented protein structure domains as a vector indicating the occurrence of fragments in the structure [20]. In both works, the maps exhibited a trend where structures formed clusters according to their fold classes, α, β, α/β, and α+β, and others, which is reasonable but expected.

Here, we present a global mapping of 3D surface shapes of single proteins and complexes. In contrast to the previous works [18–20] that considered main-chain conformation to define the structural similarity, the use of surface shape representation led to findings of previously undescribed relationships between protein shape, fold class, and assemblies. We perform a thorough analysis of surface shapes in consideration of the rise of medium- to low-resolution structures determined by electron tomography [21] and cryo-electron microscopy (cryo-EM) [22]. Classifying protein structures by shape would be more relevant to functional classes of proteins than using conventional main-chain conformations since protein functions such as binding and catalysis occur at the surfaces of proteins. As shown in our previous study [23], functionally related proteins often share similar global surface but with low sequence and backbone conformation similarity. An illustrative example is DNA topoisomerase I from human and *E*. *coli*. Despite their low sequence identity and structure similarity, both of them share a characteristic pore to encircle DNA double strand. This function similarity can be easily captured by shape descriptors, but not captured by conventional main-chain conformation approach.

Protein surface shapes were represented with 3D Zernike Descriptors (3DZD), mathematical moment-based invariants of 3D functions [23]. 3DZD has been demonstrated efficient for various biomolecular structure comparisons [24], including comparisons of EM maps [25]. Another critical difference between the current study and the previous works is that we analyzed protein complexes in comparison with single proteins. The shape mapping of single-chain and complex protein structures with 3DZD yielded a unique landscape of protein structure space that was not explored before. Dominant features that characterize protein shape are the eccentricity, which is the degree of elongation of shapes, and the number of domains. Symmetry groups are another feature that affects the shape in the case of protein complexes. A detailed analysis of the principal axis corresponding to the elongation of protein shape has suggested that proteins are required to form multimers if their shape is elongated over a certain degree. Overlapping the shape space occupied by single proteins and complexes identified shapes that are only possible in complexes. The unique view obtained from the current shape mapping leads to a more comprehensive understanding of building mechanisms, evolution, and design principles of proteins.

## Results

We first discuss the protein surface shape space for single chain proteins, followed by the analysis of shapes of protein complexes.

### Shape space of single chains

**Fig 1:**
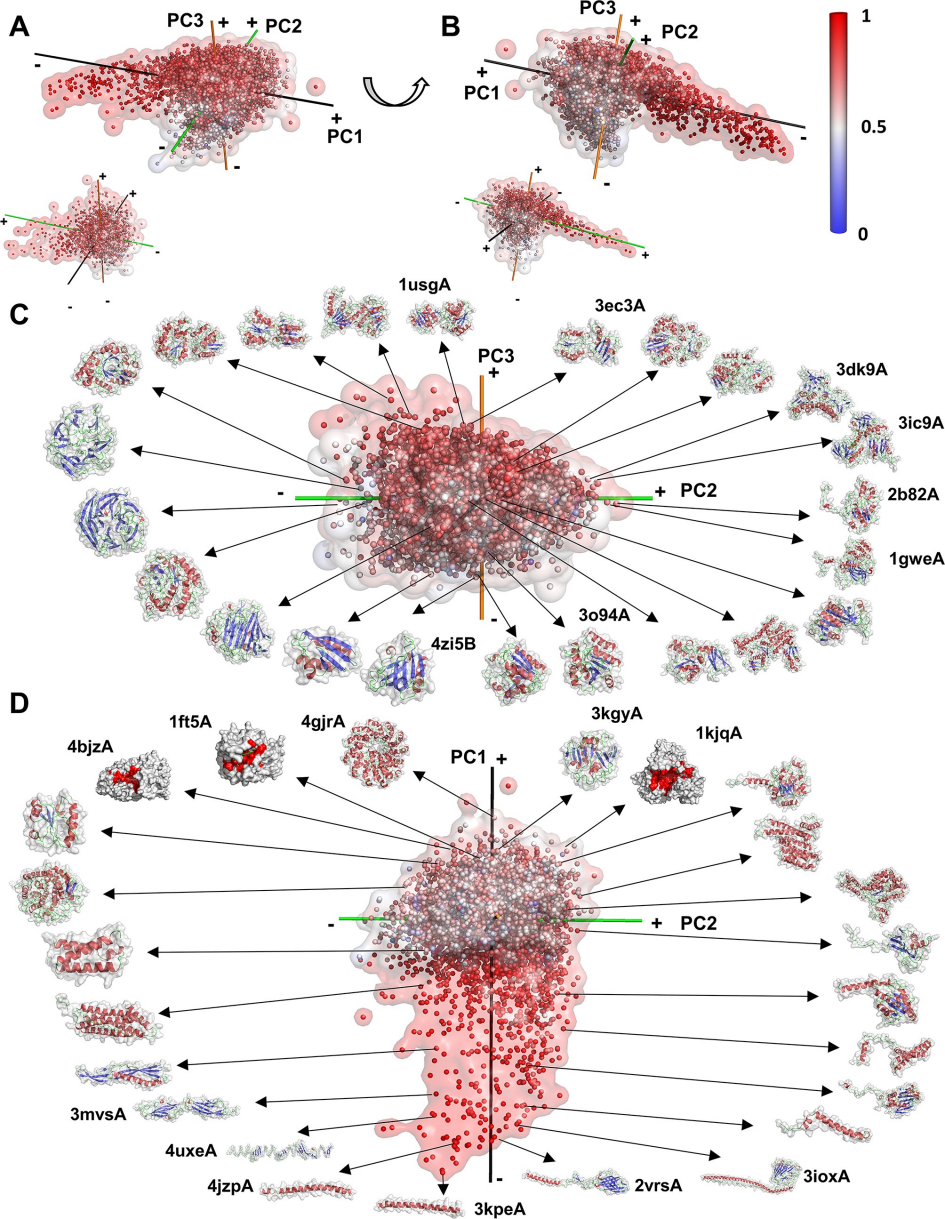
The 3D shape space of single-chain proteins. Each point corresponds to a protein. The distance between points represents the similarity of the corresponding protein shapes. The color indicates the eccentricity (the degree of elongation of a shape) from blue to red for 0.0 (sphere) to 1.0 (elongated shape). Shapes close to perfectly spherical (blue data points) do not exist in the single-chain dataset but exist in the complex structure dataset we discuss later. See Methods for the definition of the eccentricity. (A) and (B), the 3D shape space of single-chain proteins viewed from two different angles. The first, second, and third principal (PC1, PC2, and PC3) axes are shown in black, green, and orange, respectively. The positive and negative ends of an axis are labeled with + and -, respectively. The inset (a small figure of the shape space placed at bottom left) shows the distribution of high-coverage structure dataset, where a structure covers 95% or larger part of the entire protein. (C) and (D) show examples of protein shapes in the distribution on the PC2-PC3 plane (C) and on the PC1-PC2 plane (D).

Fig 1 overviews the 3D space mapping of 6,841 representative single-chain protein shapes. The surface shape of each protein was represented with the 3DZD, a rotation-invariant mathematical descriptor of 3D protein surface shape, and mapped to a 3D space using principal component analysis (PCA). 3DZD is based on a series expansion using 3D basis functions, which represents the target 3D shape by a weighted combination of the basis functions. The rotation-invariance is achieved by computing a norm of the coefficient values that are assigned to the basis functions (see Methods). PCA locates similar protein shapes close to each other in the space. The color of points indicates the eccentricity of the shapes, which quantifies how much a shape deviates from a sphere, with a higher value (red) assigned for more elongated structures (the maximum value is 1) and 0 for a perfect sphere (blue). A video clip of the 3D distribution (Appendix, S1 Movie) is also provided, along with an interactive PyMOL file (Appendix, S1 Pymol File) to help readers better understand and further investigate the 3D shape distribution.

Many entries of the Protein Data Bank (PDB) [26] contain only a fraction of the whole structure; thus, we thought it may be possible that the distribution we see in Fig 1A is biased toward surface shapes of structure fragments. For comparison, we also show in the inset figure of Fig 1A the distribution of 2,366 almost complete protein structures, which have at least 95% structure coverage of the whole proteins. The projection was made with PCA independently for this high-coverage dataset. As shown, the distribution of the high-coverage protein dataset is very similar, indicating that partial structures do not bias the distribution of the single-chain dataset.

#### Shape transition in the mapping space

**Fig 2:**
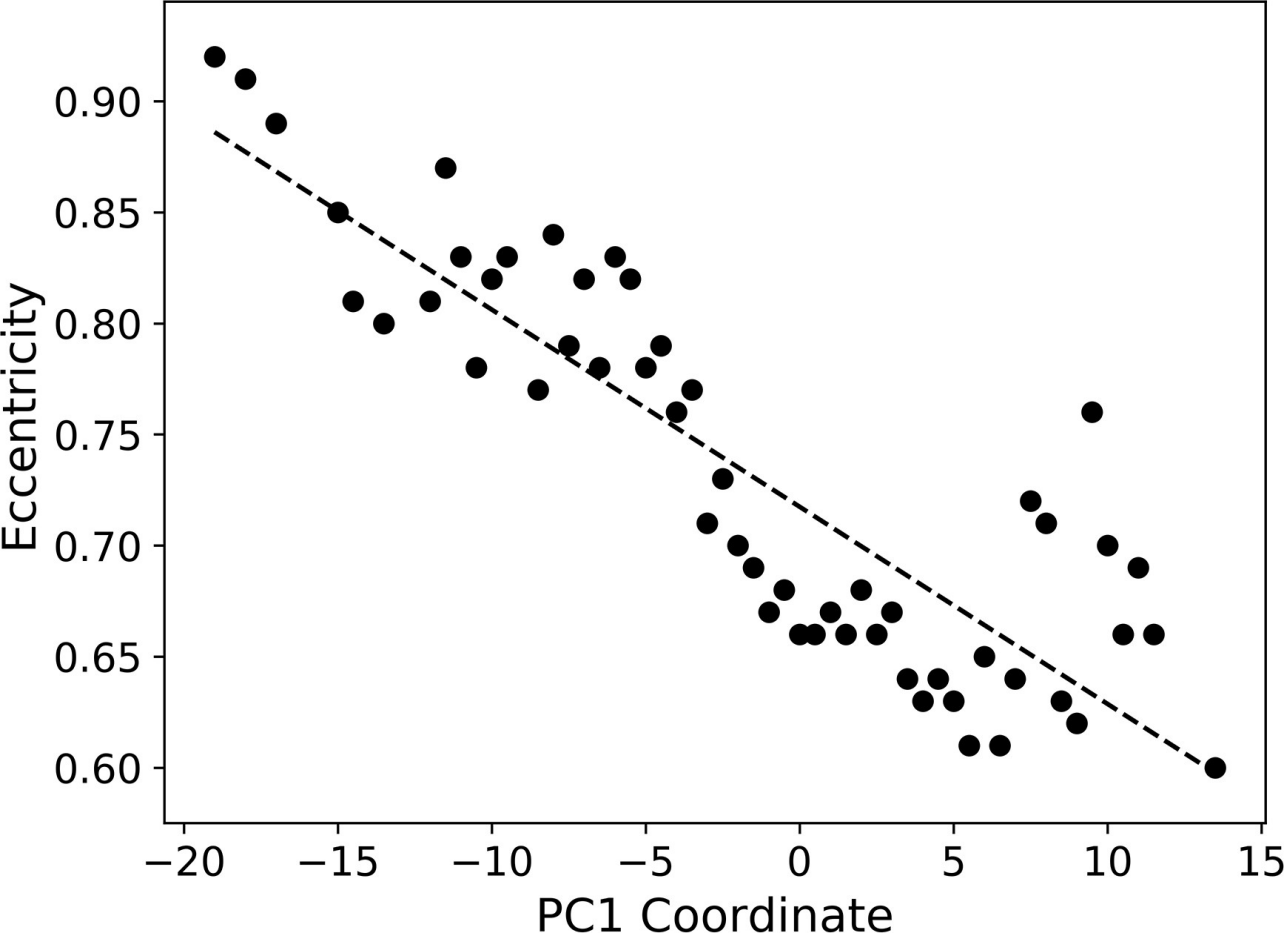
Structure transition along the first axis. The average eccentricity along the PC1 axis. Eccentricity of protein shapes are averaged at an interval of 0.5 along the axis, using shapes that locate in a sliding cylinder of a radius of 2.0 and a height of 0.5. The dashed line is the linear regression, (eccentricity) = -0.0089 * (PC1 coordinate) + 0.7174. The correlation coefficient between the eccentricity and the axis coordinate is -0.8704.

The overall distribution (Fig 1A and 1B) shows that many proteins are on or close to the plane defined by the second and the third axes (the PC2-PC3 plane) with a characteristic thin layer of “tail” region, which expands on the PC1-PC2 plane along the first axis. Proteins located in the tail region and expanded towards the negative end of the first axis have elongated shapes (colored in red). Fig 1D confirms this observation on the tail region by showing representative structures in a 2D projection. Structures located at the negative end of the first axis are single α-helices (e.g. 4jzpA, 3kpeA), which are elongated and have high eccentricity values. Next to these long α-helical proteins are proteins of elongated shapes with more secondary structure elements, including β class (e.g., 3mvsA, 4uxeA) and αβ class structures (e.g. 3ioxA, 2vrsA). On the opposite (positive) end of the first axis more spherical shapes can be found (e.g. 4gjrA, 3kgyA) colored in white to blue (Fig 1D). The average eccentricity decreases along the first axis as shown in Fig 2, which has a correlation coefficient of -0.8704. Thus, the eccentricity is the primary factor for characterizing single-chain protein shapes.

**Fig 3:**
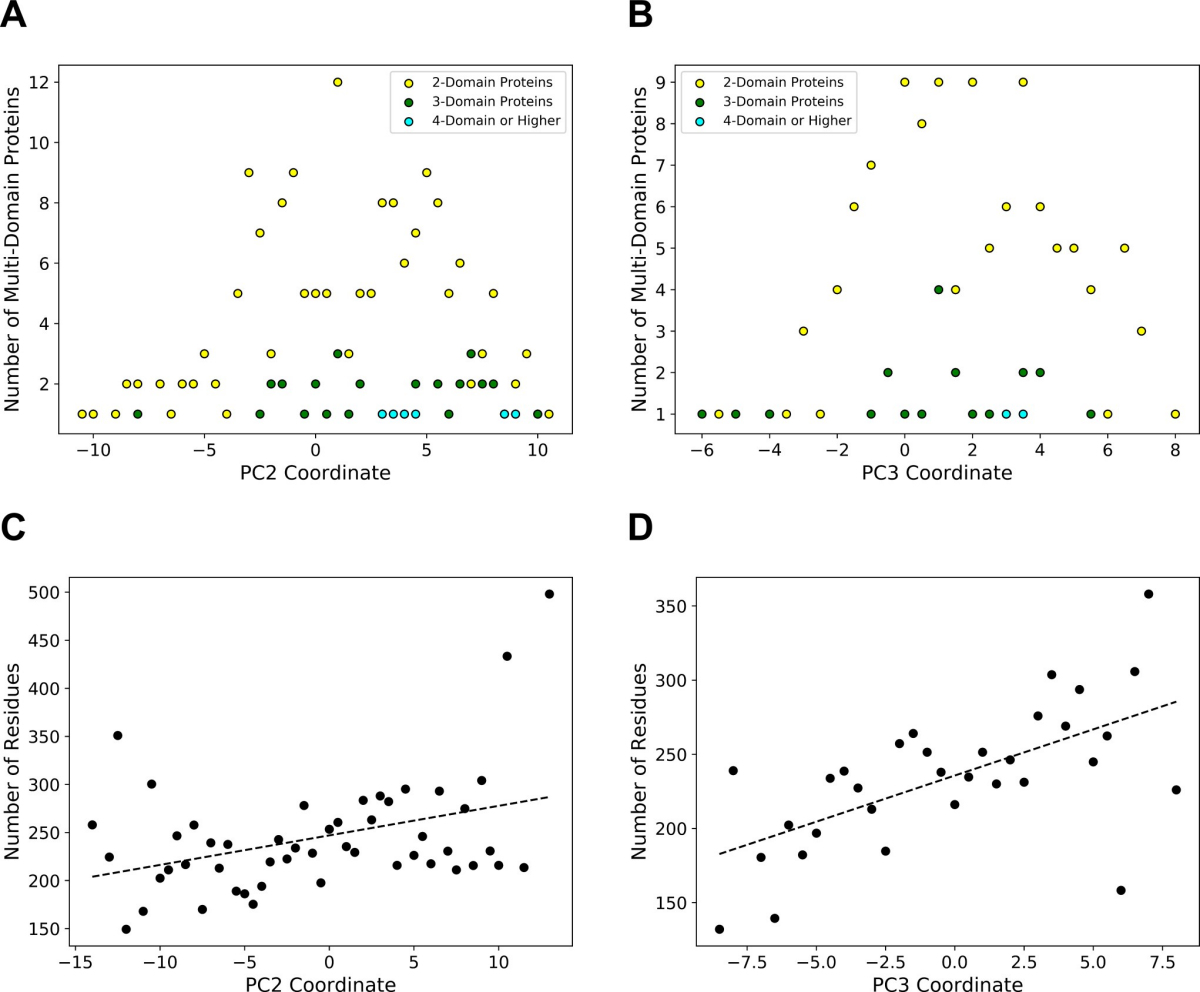
Structure transition along the second and the third axes. (A), the number of multi-domain proteins along the PC2 axis. The same sliding cylinder was used as in Fig 2. Proteins with two domains, three domains, and four or more domains are shown in yellow, green, and cyan, respectively. (B), the number of multi-domain proteins along the PC3 axis. (C), the average protein length along the PC2 axis. The same sliding cylinder was used as in Fig 2. The linear regression shown in the dashed line is (number of residues) = 3.073*(PC2 coordinate) + 246.93. The correlation coefficient is 0.3770. (D), the average protein length along the PC3 axis. The linear regression: (number of residues) = 6.226*(PC3 coordinate) + 235.61. The correlation coefficient is 0.6185.

**Fig 4:**
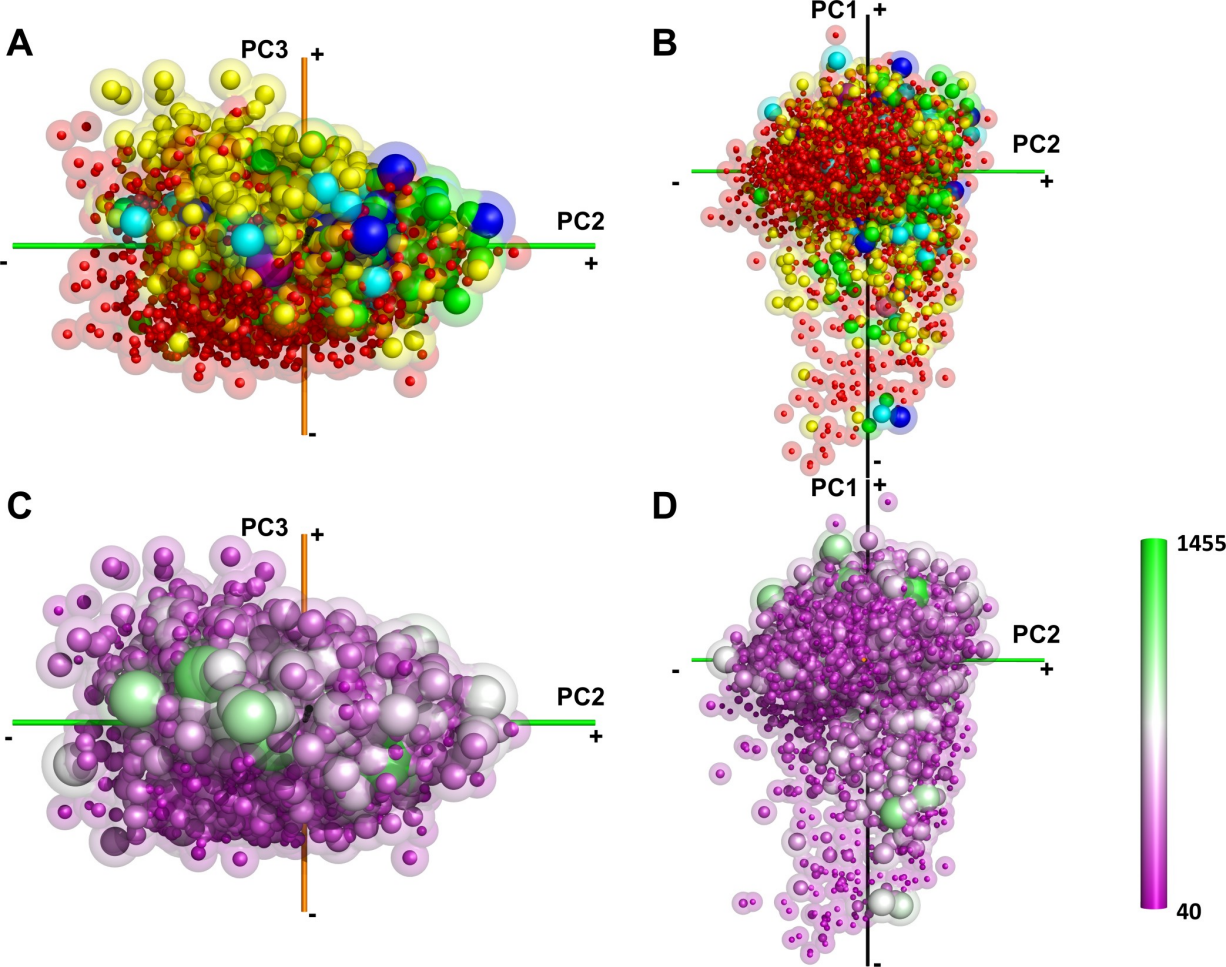
The distribution of the chain lengths and the number of domains in the single-chain shape space. (A) and (B), the number of domains in the proteins as defined by CATH. Red, yellow, green, cyan, blue, pink, and purple correspond to 1, 2, 3, 4, 5, 6, 8 domains, respectively. 6,109 proteins (89.3%) have CATH annotations. (C) and (D), the color code that ranges from purple to green shows the length (i.e. the number of amino acids) in proteins from short to long. The lengths were classified into twelve bins, 40–140, 140–240, and so on up to 1140–1540.

There are other noticeable trends in the mapping. Two-domain structures (e.g. 1usgA, 3ec3A) are dominant on the positive end of the third (orange) principal axis (Fig 1C) whereas the negative end contains more spherical single-chain shapes (e.g. 3o94A, 4zi5B). The positive end of the second (green) axis has shapes with multiple domains (e.g. 3dk9A, 3ic9A). Fig 3A and 3B plot the number of multi-domain proteins along the second axis and the third axis, respectively. The biases of observing multi-domain structures the positive side of the two axes were both statistically significant (p-value < 0.05 with χ^2^ test). Further, Fig 4A and 4B visualize the number of domains (defined in the CATH database [4]) (Fig 4A and 4B, and S2 Pymol File) in the protein shape space. Associated with the trend of multi-domain proteins in the shape space, the positive ends of the second and third axes tend to contain long proteins (Fig 4C and 4D, and S3 Pymol File). We observed weak correlations between the average protein length and the coordinates of the second and the third axes, with correlation coefficient values of 0.3770 and 0.6185, respectively (Fig 3C and 3D).

It was also observed that the positive end of the first and the second axes accumulate proteins with relatively large and deep pockets. The bias of proteins with a top 10% largest pocket being on the positive side of the two axes was statistically significant (p-value < 0.05 with χ^2^ test). Fig 1D includes three such examples, 3-hydroxybenzoate 6-hydroxylase (PDB ID: 4bjzA), which binds flavin-adenine dinucleotide (FAD), cytochrome c554 (1ft5A), which binds heme (HEM), and glycinamide ribonucleotide transformylase (1kjqA), which binds adenosine 5’-diphosphate (ADP). The pockets of these three proteins are colored in red in the figure.

Overall, proteins with similar shapes are positioned close to each other in the mapping space, and transitions of the shapes are noticeable along each axis.

#### Monomer proteins and complex-forming proteins

**Fig 5:**
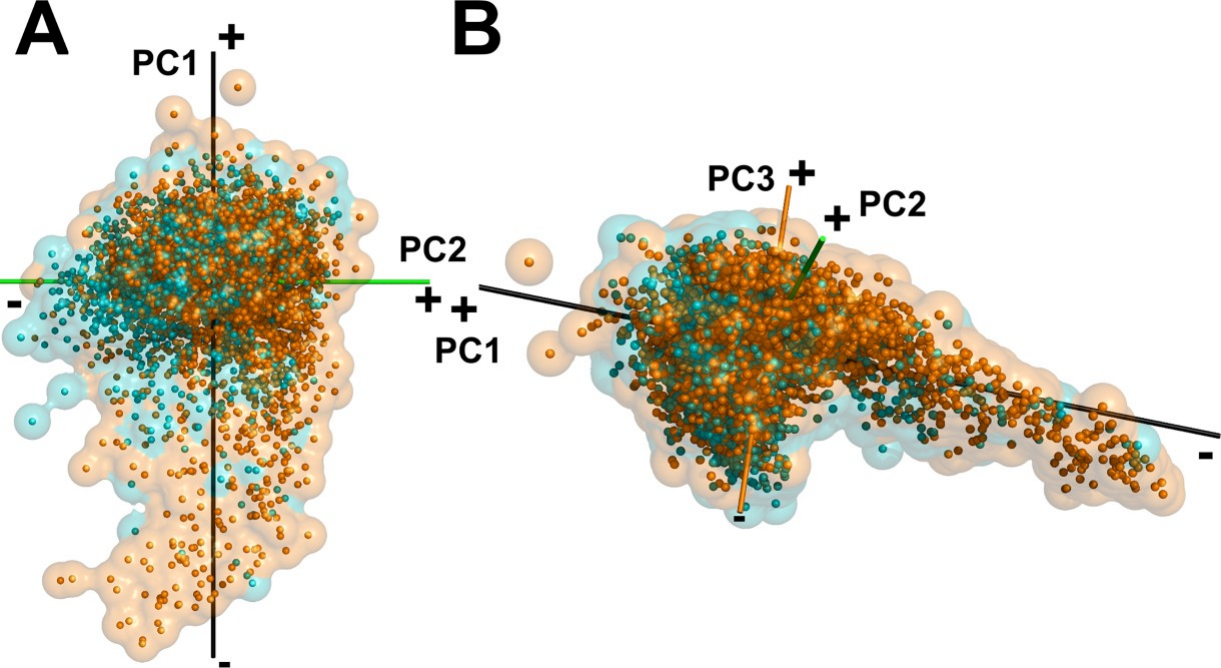
The distribution of monomers and complex forming proteins. (A), the distribution is shown on the 1–2 plane, same as the orientation in panel D in Fig 1. (B), the distribution is shown in the same orientation as panel B in Fig 1. Cyan, monomers; orange, complex-forming proteins.

A single-chain may either exist as a monomer or form a complex with other proteins in a cell. Is there any shape difference between these two classes of proteins? In Fig 5, proteins are colored in orange if they form a complex according to the biological unit information in the PISA database [27]. There are 2,259 (33.0%) monomers and 3,665 (53.6%) complex-forming proteins in the entire single-chain dataset (the remaining 13.4% do not have information in PISA). 754 (70.3%) out of 1,072 elongated-shape proteins (with an eccentricity of 0.8 or higher) are also indexed in PISA as forming complexes. On the other hand, for more spherical proteins (an eccentricity less than 0.5), the fraction of complex-forming proteins was 45.4%. The fractions of monomers and complex-formers in both elongated and spherical shapes are significantly different from the overall distribution in the entire single-chain dataset (p-value < 0.05 by χ^2^ test). Therefore, the first principal axis, which showed a gradual shift from spherical to elongated shapes, also represents the transition from monomers to complex-forming proteins.

The dataset includes 318 elongated proteins (with an eccentricity over 0.8) which PISA indicates monomers as their biological unit, not agreeing with the general trend. However, most of them (82.4%) turned out to be a part of a full structure, and if not, they interact with other proteins or nucleotides for their biological function. Examples include a ribosomal protein L22 (PDB ID: 1bxeA), which interacts with ribosomal RNAs and *Listeria monocytogenes* phage PSA endolysin (1xov) that binds to cell walls of host bacteria.

#### Protein main-chain folds in the surface mapping space

The protein fold spaces presented previously by the other groups [18–20] showed clear separation of structures of the α, β, and α/β classes in the projection space. In contrast, the protein shape space of the current work shows a very different view of the protein universe. Proteins with similar protein folds (i.e. main-chain conformations) are placed close to each other locally in the protein shape space as shown in Fig 1; however, in a larger picture there is no clear separation between different structure classes. Table 1 and S1 Fig show there are a substantial number of proteins from different classes that share similar global surface shape. Table 1 shows the number of protein pairs from various fold class combinations whose distances fall within the top closest pairs. S1 Fig shows that the 3DZD distances between proteins from different fold classes (e.g. the α class and the β class) have very similar distribution to those for protein pairs from the same fold class (e.g. both from the α class). The results imply that there may be various completely different main-chain conformations building the same protein surface shape.

**Table 1:** Structure pairs from different CATH classes in single chain dataset.

Fold class pairs	Top 0.1% *	Top 1% *	Top 5% *
α vs. α	713	5,416	23,529
β vs. β	1,035	8,849	37,581
α + β vs. α + β	6,714	63,882	311,314
SS vs. SS ^†^	5	20	54
α vs. β	1,291	12,164	54,910
α vs. α + β	2,941	29,236	146,991
α vs. SS	39	215	905
β vs. α + β	4,329	41,595	195,848
β vs. SS	9	144	738
α + β vs. SS	30	322	1,843
Others ^‡^	2,608	33,121	191,561
Total	19,714	194,964	965,274

*Number of structure pairs belonging to each specific CATH class combination within certain percentage of all structure pairs in single chain dataset. Here structure pairs are sorted by their distance in projection space, with close ones at the top.

^†^”SS” is short for “Few Secondary Structures”.

^‡^Chain with multiple domains vs. Chain with one domain or multiple domains.

### Shape space of protein complexes

**Fig 6:**
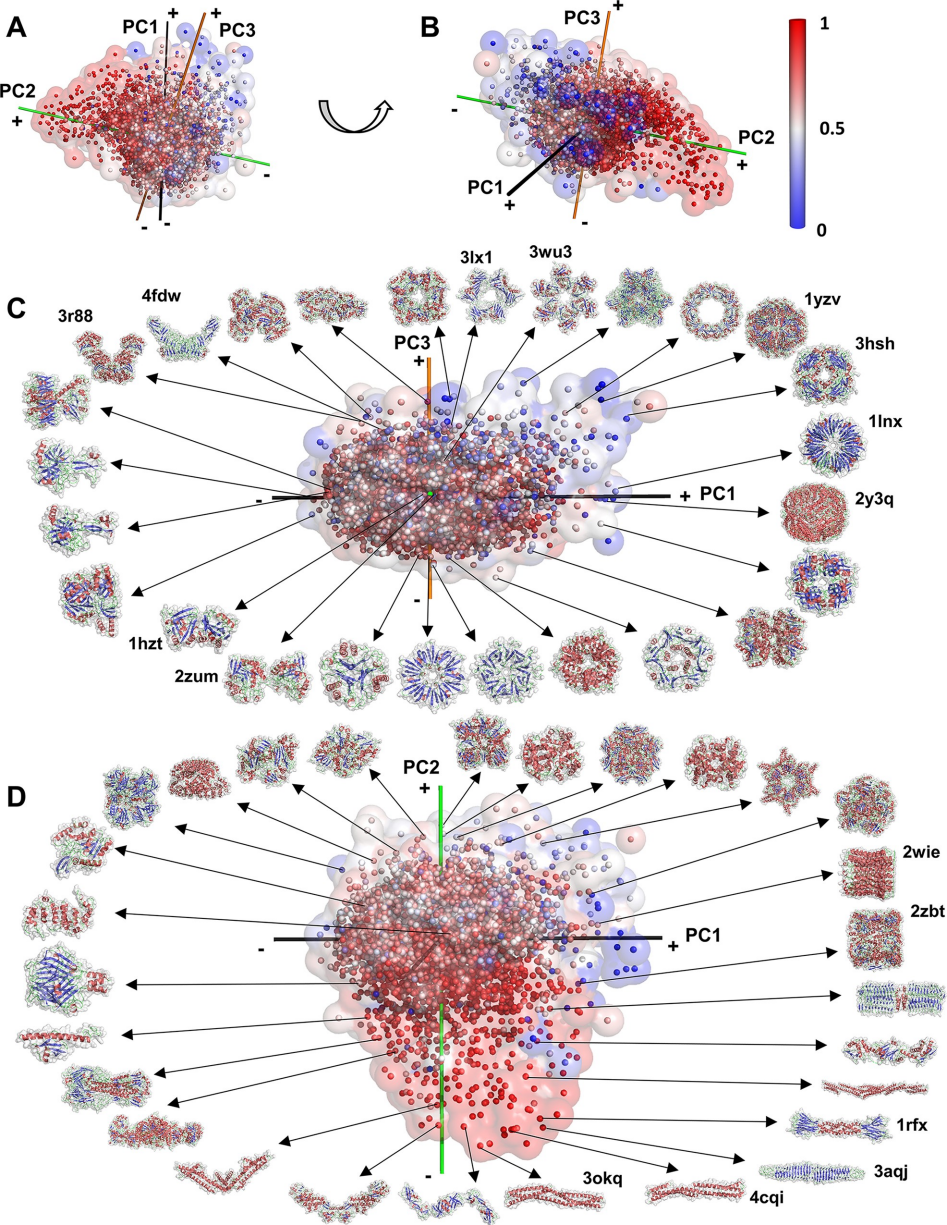
The overview of the complex shape space. 5,326 representative complex shapes are represented as points in the space. Points are colored by the eccentricity. (A) and (B), the shape space is viewed from two different angles. The color codes of axes and the eccentricity scale are the same as in Fig 1. (C) and (D) show examples of protein shapes in the distribution.

Next, we discuss the shape space of protein complexes (Fig 6, S2 Movie, S4 Pymol File). The dataset of protein complexes contains 5,326 non-redundant structures. We obtained the biological units of complexes from PISA. As in Fig 1, the color indicates the eccentricity of shapes. The complex shape space is overall very similar to the single-chain shape space (Fig 1), with the majority of structures located around the globular region near the origin of the axes and a tail region dominated by elongated shapes (the region with many red points). On the other hand, some differences were observed between the complex and single-chain distributions. The protein complexes have more spherical shapes than the single-chain distribution (data points in dark blue in the mapping) (Fig 6A and 6B). The eccentricity histograms for the single-chain and complex datasets (S2 Fig) verify this observation, which shows that the complex dataset contains highly spherical shapes with a low eccentricity. While there are no single-chain proteins with an eccentricity below 0.2, the complex dataset includes 72 such cases.

**Fig 7:**
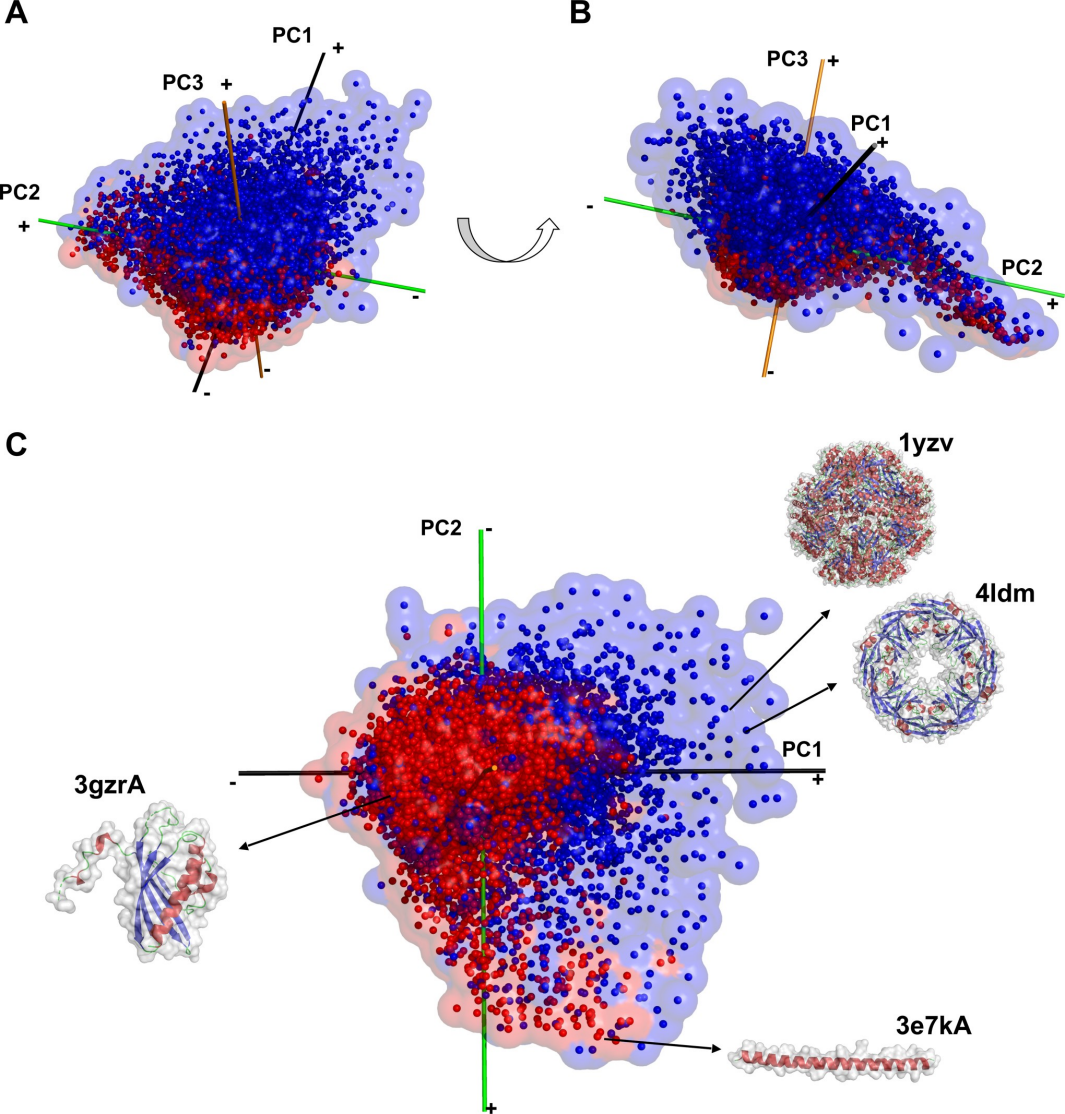
Superimposition of the single-chain and complex protein shape spaces. PCA was performed on the combination of the two datasets. Red, single-chains; blue, complex structures. (A) and (B) show the spaces in two different orientations. (C), examples of structures that locate in the single-chain specific (3e7kA and 3gzrA) and complex-structure specific (1yzv and 4ldm) areas in the protein shape space. 1yzv has octahedral symmetry and 4ldm has D4 symmetry.

The differences between the shape spaces of the single-chain proteins and complexes become apparent when they are superimposed (Fig 7, S5 Pymol File). To compare the size of the spaces occupied by the two datasets, the space was segmented into cubes of 1 axis unit edge length, and cubes were counted if they were occupied by the proteins in the datasets. Among all the cubes (3,895 cubes) that were occupied by at least one protein, 24.5% were filled by both single-chain and complex structures while 26.1% and 49.4% were occupied by only single-chain proteins and complex structures, respectively. Thus, the complex structure dataset occupies a larger space than the single-chain protein dataset. Fig 7C shows two example structures each from single-chain specific and complex-specific areas in the shape space. In the single-chain dataset, structures with a flexible tail (e.g. 3gzrA) were observed. Another example shown is 3e7kA, a narrow, elongated shape with a single helix, which is obviously very unique in single-chain proteins. On the other hand, highly spherical or symmetrical shapes are unique in protein complexes. 1yzv shown in Fig 7C has a spherical shape with the octahedral symmetry and 4ldm has a two-layer tube-like structure. The wide spread of complex structures suggests that assembling subunits into complexes can increase the range of attainable structures.

Fig 6C and 6D annotate representative structures in the complex shape space. The outskirts of the distribution in the first quadrant (i.e. top right) in Fig 6C includes shapes of the almost perfect sphere (e.g. 1yzv, 2y3q), two layers of circular ring-like arrangements (e.g. 1lnx), and cube-like shapes (e.g. 3hsh). In the second quadrant (top left) several symmetrical “spiky” shapes with multiple protrusions are observed (e.g. 4fdw, 3r88). Close to the origin (0, 0, 0), dimeric complexes (e.g. 1hzt, 2zum) are observed. Fig 6D views the complex shape mapping from a different direction, showing the tail region occupied by structures with elongated shapes. They include protein structures of different fold classes, e.g. long α helices (e.g. 4cqi, 3okq), β structures (e.g. 3aqj), mixtures of them (e.g. 1rfx), and tube-like shapes (e.g. 2wie, 2zbt).

#### Shape symmetry

**Fig 8:**
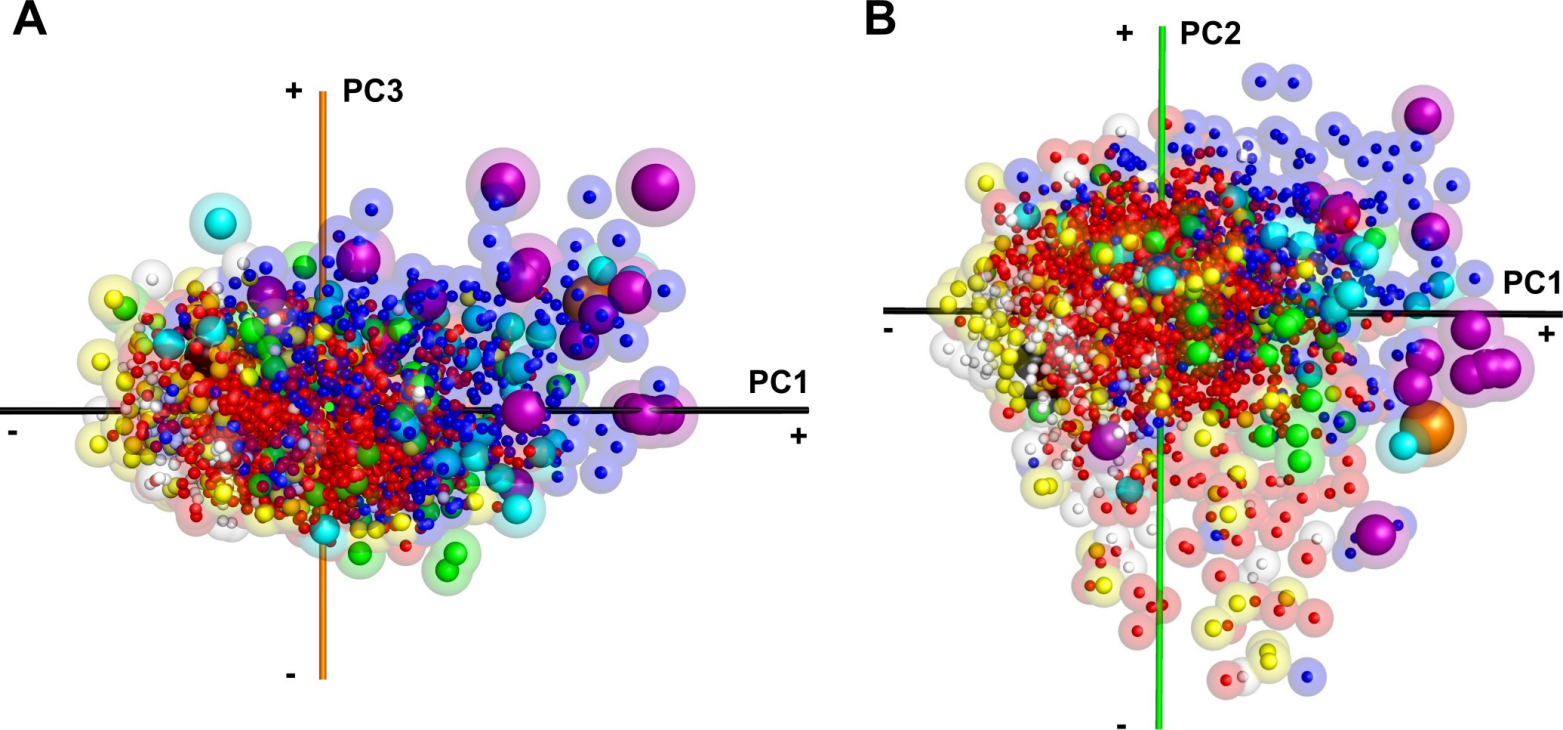
The structural symmetry of protein complexes. The protein complex shape space was colored by the structural symmetry. There were 24 symmetries in our complex dataset. Asymmetric structures, white; C2, red; C3, yellow; C4-C5, green; C6-C15, cyan. All dihedral symmetries (D2-D7) are colored in blue. Tetrahedral and octahedral, purple; icosahedral, orange; and helical, black. The radius of spheres reflects the symmetry number with a larger radius used for structures with a larger number. The distribution is shown in two orientations, A and B, which are the same as panel C and D in Fig 6.

We further examined the symmetry of complex structures (Fig 8, S6 Pymol File). Almost all complex structures have a specific symmetry type. Consistent with Fig 6C, we observed many complexes with dihedral symmetry (blue spheres, e.g. 1lnx) in the first quadrant. We also found that complexes in the first quadrant with higher-order symmetry, i.e. tetrahedral, octahedral (purple), and icosahedral symmetry (large orange sphere). These complexes are highly spherical and colored in blue in Fig 6.

#### Structures with holes

There are structures with large pockets or penetrating holes, which can be identified by comparing the volume of the structure itself and that of its convex hull (convex envelop that covers the volume). In the current analysis, buried cavities were not included because inner surface of buried cavities was not considered when the protein surface was generated. Also, tunnels in channel proteins were not effectively considered because such tunnels were too narrow to survive as cavities when the surface was constructed, and there are only three complete channel structures in the dataset in the first place.

**Fig 9:**
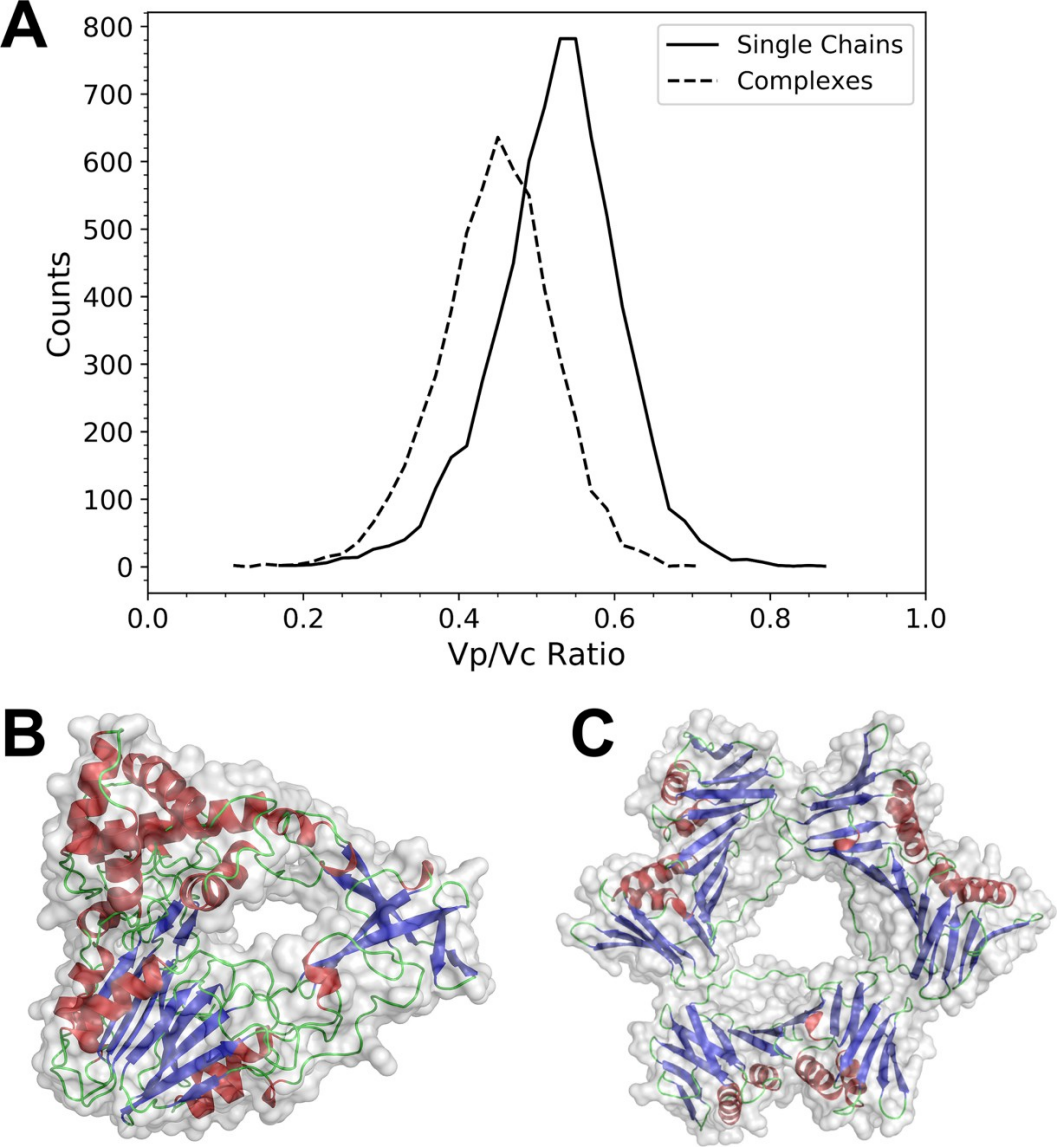
Protein structures with holes. (A) The distribution of the ratio of the protein volume (Vp) to the volume of its convex hull (Vc). Solid line, the single-chain dataset; and dashed line for the complex structure dataset. (B) glutaryl-7-aminocephalosporanic acid acylase b-chain (PDB ID: 4hst-B). The Vp/Vc Ratio is 0.408. (C) Active proliferating cell nuclear antigens (PCNAs), trimer (3lx1). The Vp/Vc ratio is 0.399.

Fig 9A shows the distribution of the ratio of the protein volume (Vp) to that of its convex hull (Vc). Complex structures tend to have a smaller Vp/Vc ratio, which is partly attributed to penetrating holes in structures. The relative abundance of structures with holes in complex structures can also be confirmed by computing a topological parameter, genus, using the Euler-Poincaré Formula. 93.1% of the single-chain structures have genus 0, which indicates that the structures do not have a hole, whereas the fraction decreases to 70.9% for complex structures. Fig 9B is an example of single-chain proteins that have large holes in the surface. The protein is a subunit of a heteromeric complex, and the holes are formed by loop regions, which provide a binding space for other subunits. Fig 9C is an example of complex shapes. It is a homo-trimeric ring-shaped complex of proliferating cell nuclear antigen (PCNA), which encircles DNA at the hole in the middle of the structure and is involved in chromosomal DNA replication [28]. In the complex dataset, there were 71 other donut-shaped complexes, which have large penetrating holes in their centers.

### Length dependency of structural features

**Fig 10:**
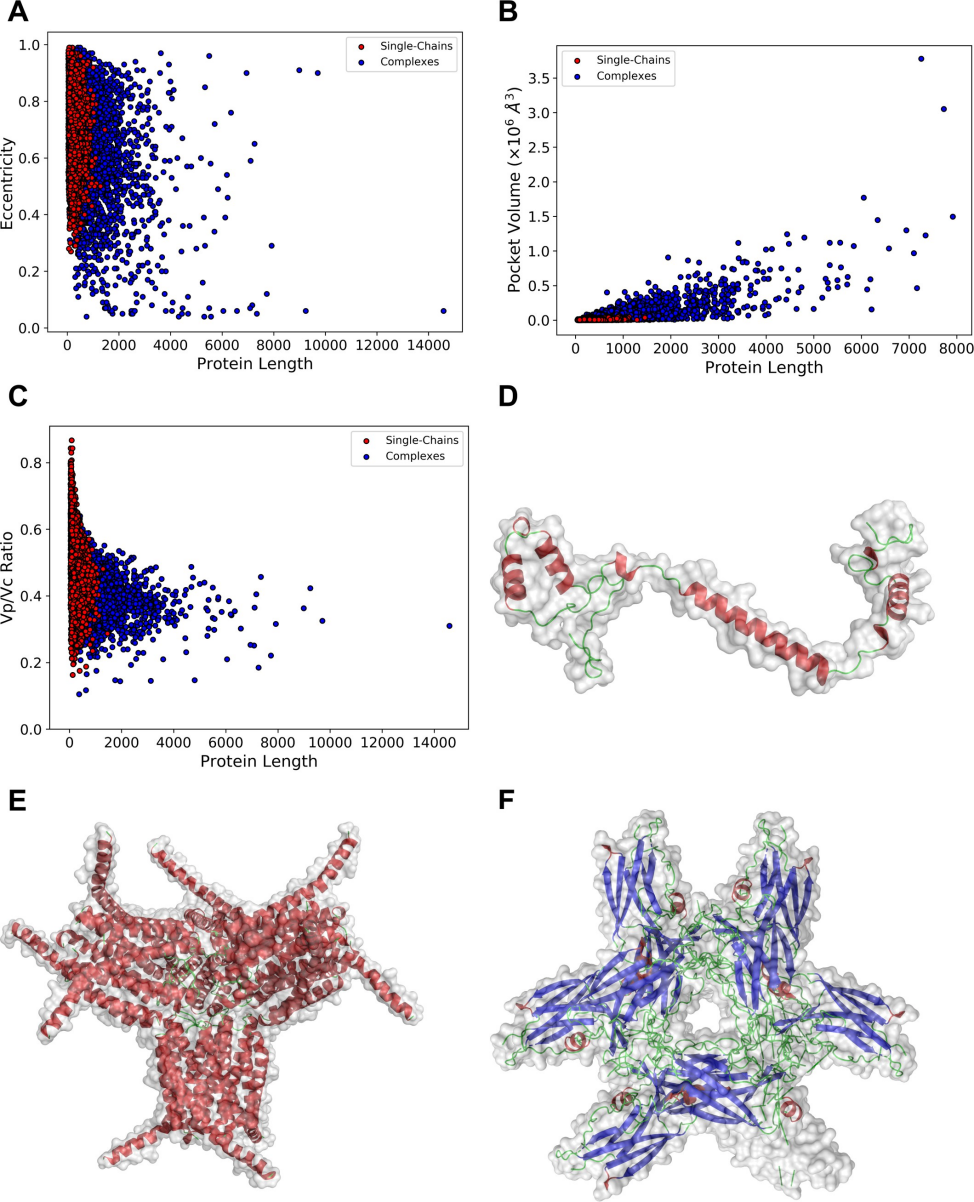
The eccentricity, the pocket size, and the Vp/Vc ratio relative to the protein length. (A), the eccentricity of proteins was plotted relative to the protein length. Red, single-chain proteins; blue, complex structures. (B), the pocket volume (Å3) relative to the protein length. (C), The Vp/Vc ratio relative to the protein length. (D), an example of single-chain proteins that have a small Vp/Vc ratio. 3ag3I, a 72 residue-long protein, which has a Vp/Vc ratio of 0.29. (E), An example of complex structures with a small Vp/Vc ratio. 3pcv, a complex with 12 chains with a total of 1,752 residues. The Vp/Vc ratio is 0.147. (F), another example of complex structures with a small Vp/Vc ratio. 3p69, a complex with 12 chains with a total of 1,524 residue long. The Vp/Vc ratio is 0.295.

In Fig 10, we examined how the eccentricity, the size of pockets, and the Vp/Vc ratio distribute relative to the number of amino acids for protein structures in the single-chain and the complex structure datasets. The first panel (Fig 10A) shows that very low eccentricity, i.e. highly spherical shapes, are achieved only by complex structures, which confirms the observation in earlier sections. Complex structures tend to have larger pockets as shown in Fig 10B. Naturally, larger protein complexes are capable of having larger pockets. Furthermore, a closer look at the plot around the protein length of up to 1,000 residues indicates that complex structures tend to have larger pockets than single-chains even when proteins of the same size are compared. Fig 10C examines the Vp/Vc ratio, the ratio of the protein volume relative to the convex hull of the protein. Overall, single-chain proteins and complex structures show similar distributions, but there are more complex structures observed in the lower end of the Vp/Vc ratio. Panels D, E, F illustrate the difference of shapes with a small Vp/Vc ratio between the two datasets. In the case of single-chains, a small Vp/Vc ratio occurs for flexible proteins such as 3ag3I (Fig 10D) while for complexes typical such shapes are symmetrical ones with protrusions (Fig 10E) and shapes with a large hollow inside as shown in Fig 10F.

## Discussion

In this study, we have constructed a mapping of the protein structure space for the first time by considering the overall surface shape of both single-chain and complex proteins. The shape space visualized in this work would give an impression that the protein shape space is continuous, but this is not specific to the protein surface shape representation. Indeed, earlier works that mapped protein structures considering main-chain conformations also show continuous structure distributions [17–20]; and moreover, there exists active discussion on the continuity [29] or the many-to-many similarity relationship [30] of the protein structure space. Analogous to well-established protein main-chain structure classifications, such as SCOP [5] and CATH [4], this work will lead to a new classification for protein shapes at a medium to low resolution, which are being accumulated at an increasing pace by cryo-electron tomography and cryo-EM. By establishing the classification from the distribution of the protein shapes, for example, we will be able to take a census of protein shapes, that is, to count the number of specific protein shapes in organisms and compare across different organisms [31].

The observed variety of protein shapes in this work will also be useful for designing protein representations used in a cell-scale physical simulation of biomolecules [32]. Rather than using an overly simplified molecular representation, as is usual for such a simulation, one could diversify protein shapes in the simulation box by sampling structures from different locations in the shape space (Fig 1 and Fig 6).

Last but not least, this work has strong implications for protein design. Our study indicates that a protein shape can be realized with utterly different backbone conformations that even belong to different fold classes as shown in Table 1 and S1 Fig. Also, the shape mappings of single chains and complexes revealed regions in the shape space that are not occupied by either of them, or are occupied only by complex shapes (Fig 7). Shapes that correspond to the former may be difficult to construct with proteins, and other materials such as DNAs or polysaccharides may be required, while those in the latter region may be better designed using complexes rather than a single-chain protein.

In the coming age of medium- to low-resolution biomolecular structures, protein design needs a novel way of viewing biomolecular shapes. We expect that this work makes a unique and significant contribution by providing a foundation of understanding the protein shape universe.

## Methods

### Single-Chain dataset

The representative set of single-chain protein structures was selected from a PISCES culled list with a resolution cutoff of 2.2 Å, an R factor cutoff of 0.2, and a pairwise sequence identity cutoff of 25% [33]. From 7,260 chains in the list, we removed short chains with less than 40 amino acids. We have also removed proteins that have a large spatial gap, i.e. structures having more than one cluster when C_α_ atoms were clustered with a 9 Å cutoff. We further removed 82 chains were further removed from the list because their sequences had more than 25% sequence identity to other chains. This process yielded a dataset of 6,841 non-redundant protein structures.

From this dataset, we prepared another dataset by pruning structures that include less than 95% of residues relative to the whole chain length. The protein lengths were obtained from UniProt [34]. There are 2,366 chains in this high-coverage single chain dataset. For each chain, fold class was assigned following CATH. Also, by referring to PISA [27], we assigned biological unit information. This pruned dataset was shown in inset of Fig 1A and 1B.

### Complex dataset

From PDB, we identified structures that exist as a complex as defined in PISA and downloaded the first biological unit (BU). The same resolution, R factor, and length cutoffs as in the single chain dataset were applied. A complex is considered as redundant if there is another complex with the same number of chains and corresponding chains between them have over 25% sequence identity. Among redundant complex entries, we chose the one with the highest resolution and the lowest R factor. This procedure yielded 5,326 complexes. Symmetry information for complexes was obtained from PDB if the BU of the complex considered has the same composition as in PDB. Out of the 5,326 complexes, 2,876 of them acquired symmetry information.

### Protein surface shape representation

We used 3DZD, mathematical rotation-invariant moment-based descriptors, to represent the surface shape of single-chain proteins and complexes. For a protein structure, a surface was constructed using the MSMS program [35] and then mapped to a 3D cubic grid of the size of N^3^ (N was set to 200). Protein size is not explicitly considered in 3DZD calculation. But in our previous study [23], we have shown that it is rare for proteins with very different sizes to share similar global surface. Moreover, in Fig 4C and 4D, we have also analyzed the chain length distribution in the single-chain shape space. MSMS failed to generate surface for two cases each in the single-chain dataset and the complex structure dataset, for which we used the MSROLL program [36] instead. Each voxel (a cube defined by the grid) is assigned either 1 or 0; 1 for a surface voxel that locates closer than 1.7 grid interval to any triangle defining the protein surface, and 0 otherwise. This 3D grid with 1s and 0s was considered as a 3D function *f*(**x**), for which a series is computed in terms of the Zernike-Canterakis basis [37] that is defined by the collection of functions

$$Z_{nl}^{m}(r,\vartheta ,\varphi )=R_{nl}(r)Y_{l}^{m}(\vartheta ,\varphi )$$ (1)

with −*l*<*m*<*l*, 0≤*l*≤*n*, and (*n*−*l*) even. $Y_{l}^{m}(\vartheta ,\varphi )$ are spherical harmonics. *R*_*nl*_(*r*) are radial functions defined by Canterakis, constructed so that $Z_{nl}^{m}(r,\vartheta ,\varphi )$ are homogeneous polynomials when written in terms of Cartesian coordinates. 3D Zernike moments of *f*(**x**) are defined as the coefficients of the expansion in this orthonormal basis, *i*.*e*. by the formula

$$\Omega_{nl}^{m}=\frac{3}{4\pi }\int_{\left|x\right|\leq 1}f(x){\bar{Z}}_{nl}^{m}(x)dx.$$ (2)

To achieve rotation invariance, the moments are collected into (2*l*+1)-dimensional vectors $\Omega_{nl}=(\Omega_{nl}^{l},\Omega_{nl}^{l-1},\Omega_{nl}^{l-2},\Omega_{nl}^{l-3},\ldots ,\Omega_{nl}^{-l})$, and the rotationally invariant 3D Zernike descriptors *F*_*nl*_ are defined as norms of the vectors Ω_*nl*_. Thus

$$F_{nl}^{}=\sqrt{\sum_{m=-l}^{m=l}{\left(\Omega_{nl}^{m}\right)}^{2}}$$ (3)

Index *n* is called the order of the descriptor. The rotational invariance of 3D Zernike descriptors means *e*.*g*. that calculating *F*_*nl*_ for a protein and its rotated version would yield the same result. We used 20 as the order because it gave reasonable results in our previous works on protein 3D shape comparison [23,38–40]. A 3DZD with an order *n* of 20 represents a 3D structure as a vector of 121 invariants [23]. The similarity between two proteins X and Y was measured by the Euclidean distance *d*_*E*_ between their 3DZDs, $d_{E}=\sqrt{\sum_{i=1}^{121}{\left(X_{i}-Y_{i}\right)}^{2}}$, where *X*_*i*_ and *Y*_*i*_ represent the *i*th invariant for protein X and Y, respectively.

To illustrate the characteristics of 3DZDs, we compare it against two other structure similarity measures, the Procrustes distance [41] and TM-Score [42]. The Procrustes distance is a root-mean square deviation (RMSD) between corresponding points in two objects after an appropriate optimization of translation, rotation, and scaling. The smaller the Procrustes distance, the more similar the shape are. On the other hand, TM-Score is one of the common measures of the similarity of the main-chain conformations of proteins. TM-Score ranges from 0 to 1, with 1 for identical protein structures. Proteins within the same fold usually have a score above 0.5. The Euclidean distance of 3DZD is usually below 10 for proteins of the same shape [23,39].

In S3 Fig, the Euclidian distance of 3DZD and the Procrustes distance were compared in two datasets. Panel A compares pairs of 20 ellipsoids with increasing eccentricities, while panel B shows results on 1,278 single-chain protein pairs that have the same number of vertices in the surface representation. The two measures correlated well with a correlation coefficient of 0.9784 for the ellipsoid dataset (S3A Fig), because surface points were systematically distributed in the same fashion for all the ellipsoids and thus corresponding points are easily matched for aligning two ellipsoids. On the other hand, the two measures often have very different distances in protein shape cases (S3B Fig), which typically happened when point correspondences do not even allow appropriate scaling of the two structures. In S3B Fig, there are many protein pairs that have different surface shapes with a 3DZD Euclidean distance of over 10 but with a small Procrustes distance of around 0.2. S3C and S3D Fig show such protein pairs. As shown, proteins in these pairs have very different shapes, which indicates that 3DZD performs more reasonably for comparing protein shapes. Indeed, for protein shape comparison, The Procrustes distance has difficulty because corresponding surface points in two proteins need to be determined prior to the distance computation, which are not available in general for protein surface comparison. This is more difficult when two proteins have a different number of surface points to be compared. Apparently, 3DZD does not have such a problem because it does not align points to points.

S4A and S4B Fig show the comparison between 3DZD and TM-Score. As shown, these two measures have virtually no correlation. The correlation coefficient was -0.1735 for these two measures. Panel B shows the density of the two measures. The highest density (yellow) was observed at around 3DZD distance of 5 to 10 and TM-score of 0.3, which is the score range for proteins with similar surface shape but with different main-chain fold. As also shown in Table 1, there are cases that proteins of the different fold class have a small 3DZD Euclidian distance. S4C and S4D Fig shows two such examples, where two structures have a similar surface shape to each other according to 3DZD but have a very large difference in their main-chain conformations. These results are consistent with our earlier work where we extensively compared 3DZD with conventional protein structure comparison methods [23].

The 3DZD files of the single-chain and the complex datasets are made available at S1 Data. 3DZD can be also computed for PDB files at the benchmark page of 3D-SURFER (http://kiharalab.org/3d-surfer/batch.php) [25,38].

### Mapping structures

We used principal component analysis (PCA) to project 3DZDs of 121 value vectors of protein structures into 3D. Three eigenvectors were chosen for the mapping because the scree plots (S5 Fig) showed that adding more eigenvalues does not contribute much to explaining data variance, and also to be consistent with the previous related works [18–20]. The three eigenvalues explained 52.64% and 47.76% of the total variation in the single-chain and the complex structure datasets, respectively.

### Eccentricity of a protein shape

In order to quantify how elongated a structure is, we have defined the term eccentricity, which is calculated from the minimum volume enclosing ellipsoid (MVEE) of a structure. Given all atoms in a structure, protein MVEE is the ellipsoid with minimum volume that encloses all atoms. From MVEE, the eccentricity is defined as $\sqrt{\left(2-b^{2}/a^{2}-c^{2}/a^{2}\right)/2}$, where *a*, *b*, and *c* are the length of longest, the second longest, and the third longest semi-principal axes of the ellipsoid, respectively. Elongated structures have an eccentricity close to 1, while spherical structures have an eccentricity close to 0.

### Protein volume computation

The volume of proteins was computed using MSROLL with a probe radius set to 0. For 42 cases in the single-chain dataset and 82 cases in the complex dataset where the MSROLL failed, we used the ProteinVolume program [43] instead. The volume values computed by these two programs were very consistent; the difference of volume values for ten randomly selected protein structures was on average 1.04%. The convex hull of a protein structure and its volume was computed using the ConvexHull function in the scipy.spatial package [44].

A pocket on a protein surface was identified and its volume was computed with VisGrid [45]. The average size of the pocket volume in the single-chain proteins was 6,302.9 Å^3^. We analyzed the location of proteins with a large pocket whose size is within the top 10% (12,219 Å^3^ or larger) in the single-chain protein surface space (Fig 1D).

### The genus number

Donut-shaped structures were identified by first screening structures with genus > 0 and then with the conditions of 0.9≤*b*/*a*≤1.0 and $0\leq \sqrt{{(c}^{2}/a^{2}+c^{2}/b^{2})/2}\leq 0.6$, where *a*, *b*, and *c* are the parameters of MVEE of the structures. Then, structures that passed the criteria were visually examined. The genus number was computed with the Euler-Poincaré Formula, which states the following relationship between the number of vertices (V), edges (E), faces (F), loops (L), shells (S), and genus (g) of a manifold: V + F–E–(L–F) = 2 (S–g). To obtain these values of a protein surface, we used triangular meshes computed by EDTSurf [46]. L is equal to F for triangle meshes since triangular faces have exactly 1 loop. S was computed as the number of disconnected groups of faces.

## Supporting information

S1 Fig Distribution of 3DZD distances of protein pairs from different fold classes in the single-chain protein dataset. Top, the histogram of the 3DZD distances of proteins from different combinations of fold classes. Fold class information was obtained from the CATH database. The y-axis shows the fraction of pairs that falls into each distance bins. Two peaks are observed for pairs that involve the few secondary structure (ss) class. There are only 28 chains in the few ss class. Those chains have roughly two kinds of shapes, either elongated, or relatively spherical. The peak at a relatively small distance corresponds to pairs within each category, while the peak at a relatively large distance corresponds to pairs across two categories. Bottom, the 3DZD distance distribution of up to a bin of 4.0–5.0. The y-axis is now the actual number of protein pairs.
(PDF)

S2 Fig Histograms of eccentricity of the single-chain and complex datasets. The blue line is for the single-chain protein dataset, while the orange line is for complex dataset.
(PDF)

S3 Fig Comparison between 3DZD and the Procrustes distance. (A), Comparison of the Euclidian distance of 3DZD and the Procrustes distance for all the pairs of 20 ellipsoids with increasing eccentricity values from 0.0 to 0.92. On each ellipsoid 2500 points were sampled uniformly on the spherical coordinates. The two angles, θ (0 to π) and φ (0 to 2π) were evenly divided into 50 intervals and a point was placed on the ellipsoid surface for each combination of θ and φ. (B), The 3DZD and the Procrustes distances were compared for 1,278 single-chain protein pairs that have the same number of vertices in the surface triangle mesh representation. For computing the Procrustes distance for a protein pair, the closest surface point pairs from the two proteins were matched using the coherent point draft algorithm. (C), an example of protein pairs that have a large 3DZD distance and a small Procrustes distance. 2bwrA (CATH code: N/A) and 3ke3A (CATH: 3.40.640.10, 3.90.1150.10, there are two CATH codes because this is a two-domain structure). The 3DZD distance: 13.88; the Procrustes distance: 0.19. (D), another such example of protein pairs. 4gnrA (CATH: 3.40.50.2300, 3.40.50.2300) and 3ga7A (CATH: 3.40.50.1820). The 3DZD distance: 13.13; the Procrustes distance: 0.18.
(PDF)

S4 Fig Comparison between 3DZD and the TM-Score on the single-chain dataset. (A), each point represents a protein pair. (B), the same data are represented with the density information. (C), an example of protein pairs that has a small 3DZD Euclidian distance but from different fold classes, the α class and the β class. Left, PDB ID: 1c3cA; CATH code: 1.10.276.10. Right, 4jp0A, 2.80.10.50. The Euclidian distance of 3DZD was 2.4, while the TM-score was 0.265. (D), another example of protein pairs with a small 3DZD Euclidian distance but from different fold classes, the β class and the αβ class. Left, 3a6rA; 2.30.110.10. Right, 3h87A, 3.40.50.1010. The Euclidian distance of 3DZD was 2.4, while the TM-score was 0.254.
(PDF)

S5 Fig Scree plots of single-chain and complex datasets. The figure shows top 10 eigenvalues of the covariance matrix sorted in the descending order. The insert shows all 121 eigenvalues. Eigenvalues of single-chain, high-coverage single-chain and complex datasets are colored in red, orange and blue, respectively. The sharp drop up to the third eigenvalue indicates that adding fourth and more eigenvalues do not add substantially more information.
(PDF)

S1 Data A zipped file of 3DZD files of the single-chain and complex datasets. (ZIP)

S1 Movie Overview of the 3D shape space of single-chain proteins. Same as the representations used in Fig 1, each point corresponds to a protein. The point color indicates the eccentricity from blue to red for 0.0 (sphere) to 1.0 (elongated shape). The first, second and third axes are shown in black, green and orange, respectively. Orientations of the shape space shown in this movie are in the following order: Fig 1A -> x-y plane from z(+) -> x-z plane from y(+) -> Fig 1D -> x-z plane from y(-) -> Fig 1A -> Fig 2A -> Fig 1A.
(MOV)

S2 Movie Overview of the 3D shape space of complexes. Same as the representations adopted in Fig 6, each point corresponds to a complex. The point color indicates the eccentricity from blue to red for 0.0 (sphere) to 1.0 (elongated shape). The first, second and third axes are shown in black, green and orange, respectively. Orientations of the shape space shown in this movie are in the following order: Fig 6A -> Fig 6C -> x-y plane from z(+) -> x-z plane from y(+) -> Fig 6D -> Fig 6A.
(MOV)

S1 Pymol File The 3D shape space of single-chain proteins shown in the molecular visualization software Pymol. Pymol is a 3D biomolecular structure viewer and freely available at https://pymol.org/2/. Readers can download the Pymol software and open the session file in Pymol to rotate the shape space in 3D. Same as Fig 1 and S1 Movie, each single-chain protein is represented as a point colored by the eccentricity value.
(PSE)

S2 Pymol File The distribution of the number of domains in the single-chain shape space shown in the molecular visualization software Pymol. The coloring of the points follows the same pattern as Fig 4A and 4B. Red, yellow, green, cyan, blue, pink, and purple correspond to 1, 2, 3, 4, 5, 6, 8 domains, respectively.
(PSE)

S3 Pymol File The distribution of the chain lengths in the single-chain shape space shown in the molecular visualization software Pymol. The coloring of the points follows the same pattern as Fig 4C and 4D. The color code that ranges from purple to green shows the length (i.e., the number of amino acids) in proteins from short to long. The lengths are classified into twelve bins, 40–140, 140–240, and so on up to 1140–1540.
(PSE)

S4 Pymol File The 3D shape space of complexes shown in the molecular visualization software Pymol. The color code is the same as the one used in Fig 6 and S2 Movie. Each complex is represented as a point colored by the eccentricity value.
(PSE)

S5 Pymol File Superimposition of the single-chain and complex protein shape spaces shown in the molecular visualization software Pymol. Red points represent single-chain proteins. Blue points represent complexes. This file corresponds to Fig 7.
(PSE)

S6 Pymol File The structural symmetry of protein complexes shown in the molecular visualization software Pymol. The color code is the same as the one used in Fig 8. Asymmetric structures, white; C2, red; C3, yellow; C4-C5, green; C6-C15, cyan. All dihedral symmetries (D2-D7) are colored in blue. Tetrahedral and octahedral, purple; icosahedral, orange; and helical, black. The radius of spheres reflects the symmetry number with a larger radius used for structures with a larger number.
(PSE)
