# Supplementary figures and images for: A global map of the protein shape universe
Source: PLoS Comput Biol. 2019 Apr 12;15(4):e1006969. doi: 10.1371/journal.pcbi.1006969 (PMC6481876; doi:10.1371/journal.pcbi.1006969)

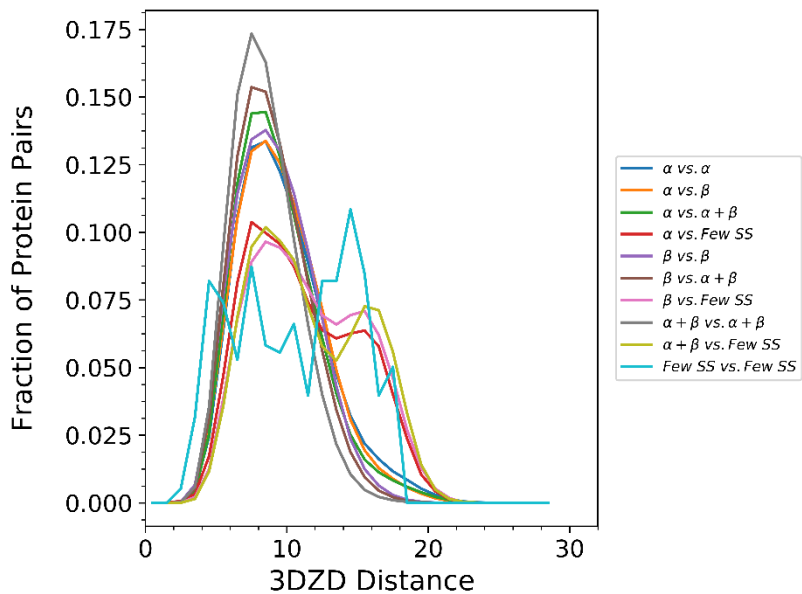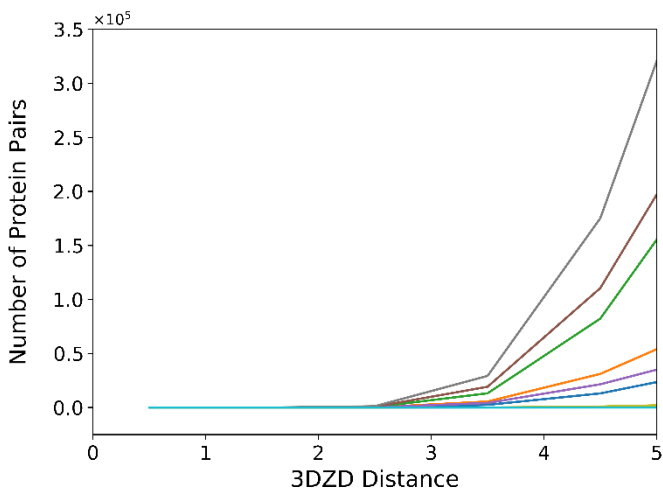

Supplement: S1 Fig — Top, the histogram of the 3DZD distances of proteins from different combinations of fold classes. Fold class information was obtained from the CATH database. The y-axis shows the fraction of pairs that falls into each distance bins. Two peaks are observed for pairs that involve the few secondary structure (ss) class. There are only 28 chains in the few ss class. Those chains have roughly two kinds of shapes, either elongated, or relatively spherical. The peak at a relatively small distance corresponds to pairs within each category, while the peak at a relatively large distance corresponds to pairs across two categories. Bottom, the 3DZD distance distribution of up to a bin of 4.0–5.0. The y-axis is now the actual number of protein pairs. (PDF) [file pcbi.1006969.s001.pdf]

Number of Structures

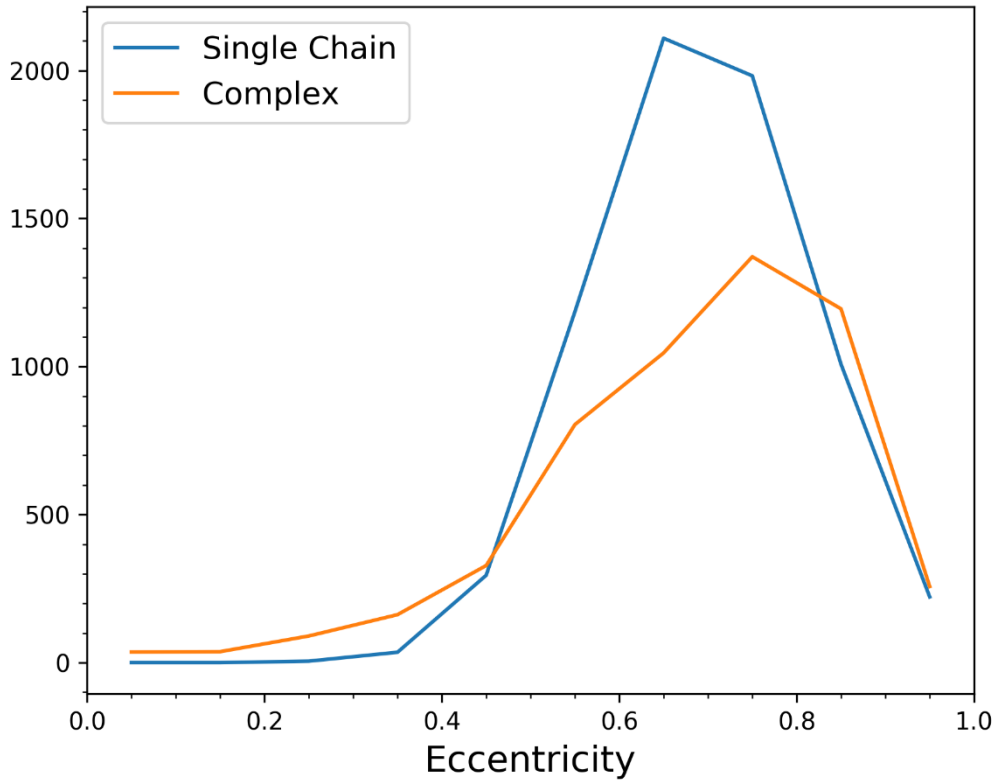

Supplement: S2 Fig — The blue line is for the single-chain protein dataset, while the orange line is for complex dataset. (PDF) [file pcbi.1006969.s002.pdf]

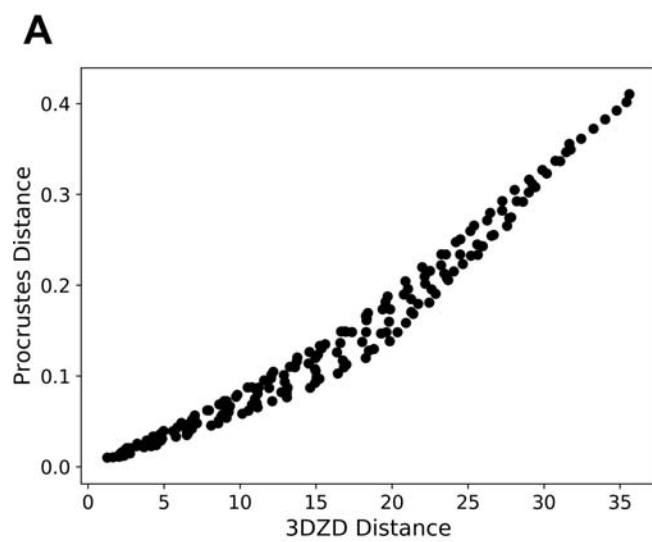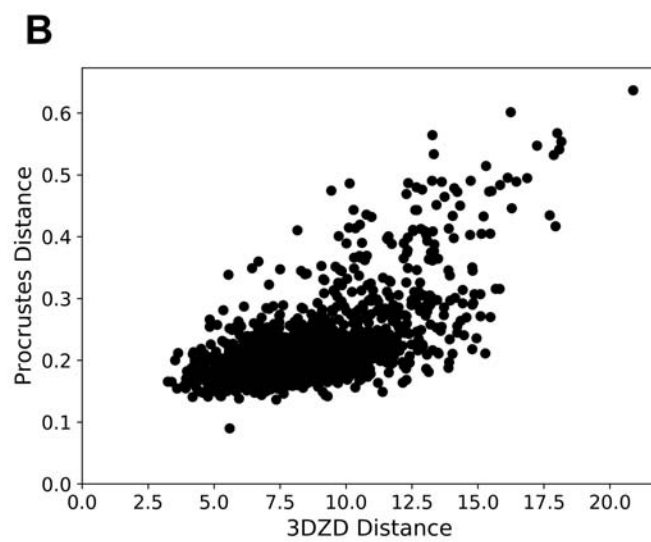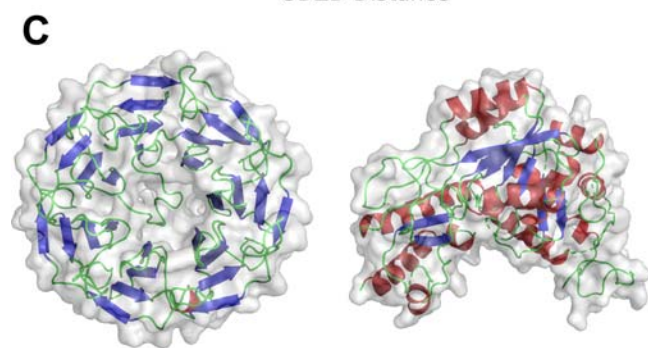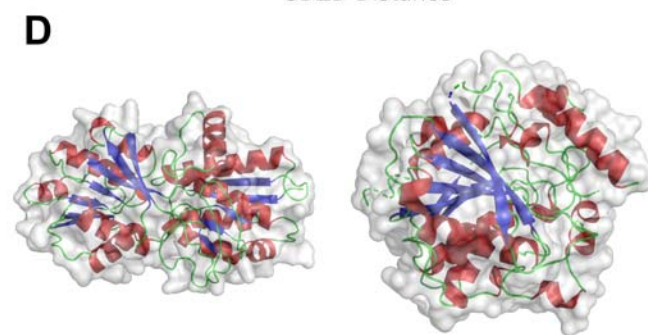

Supplement: S3 Fig — (A), Comparison of the Euclidian distance of 3DZD and the Procrustes distance for all the pairs of 20 ellipsoids with increasing eccentricity values from 0.0 to 0.92. On each ellipsoid 2500 points were sampled uniformly on the spherical coordinates. The two angles, θ (0 to π) and φ (0 to 2π) were evenly divided into 50 intervals and a point was placed on the ellipsoid surface for each combination of θ and φ. (B), The 3DZD and the Procrustes distances were compared for 1,278 single-chain protein pairs that have the same number of vertices in the surface triangle mesh representation. For computing the Procrustes distance for a protein pair, the closest surface point pairs from the two proteins were matched using the coherent point draft algorithm. (C), an example of protein pairs that have a large 3DZD distance and a small Procrustes distance. 2bwrA (CATH code: N/A) and 3ke3A (CATH: 3.40.640.10, 3.90.1150.10, there are two CATH codes because this is a two-domain structure). The 3DZD distance: 13.88; the Procrustes distance: 0.19. (D), another such example of protein pairs. 4gnrA (CATH: 3.40.50.2300, 3.40.50.2300) and 3ga7A (CATH: 3.40.50.1820). The 3DZD distance: 13.13; the Procrustes distance: 0.18. (PDF) [file pcbi.1006969.s003.pdf]

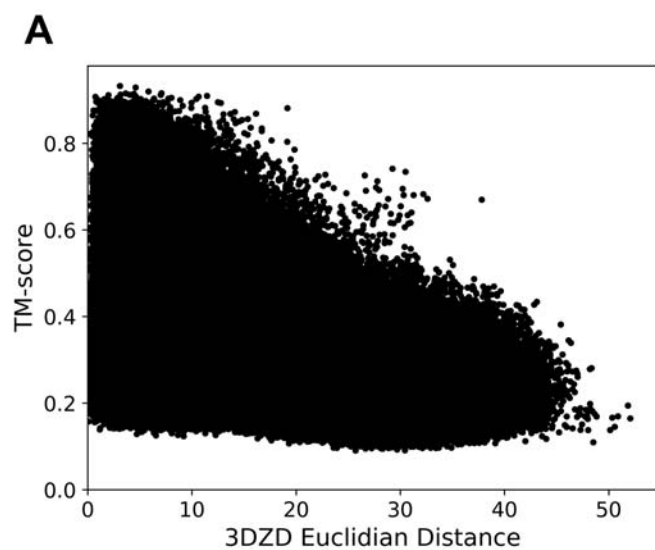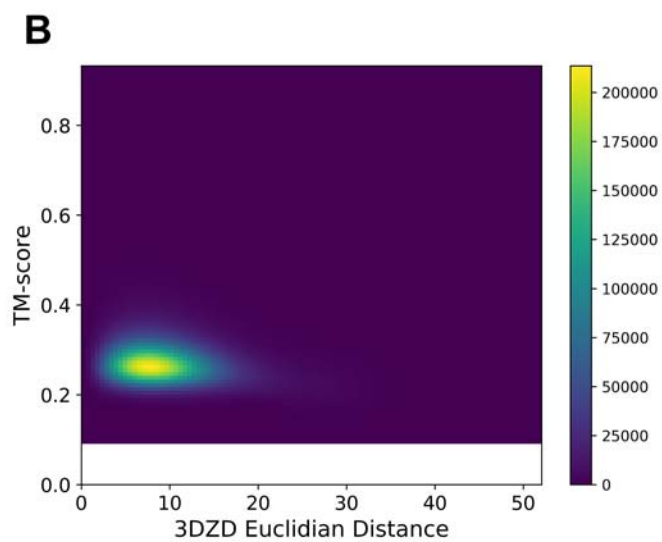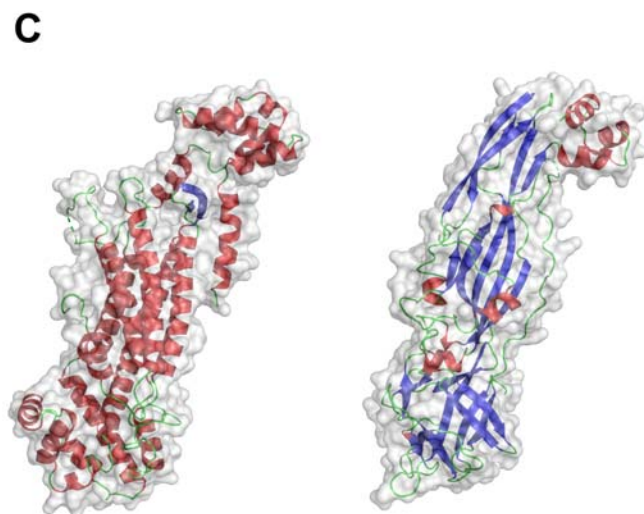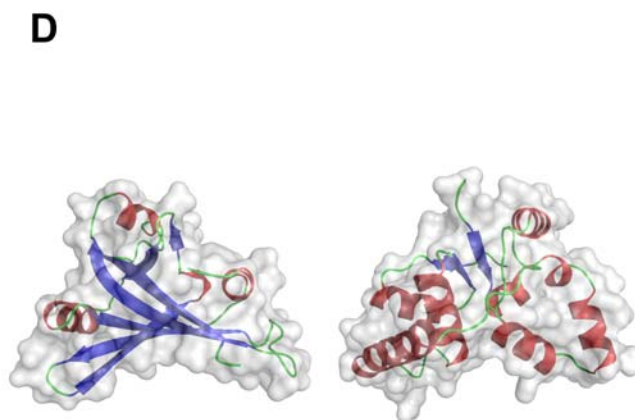

Supplement: S4 Fig — (A), each point represents a protein pair. (B), the same data are represented with the density information. (C), an example of protein pairs that has a small 3DZD Euclidian distance but from different fold classes, the α class and the β class. Left, PDB ID: 1c3cA; CATH code: 1.10.276.10. Right, 4jp0A, 2.80.10.50. The Euclidian distance of 3DZD was 2.4, while the TM-score was 0.265. (D), another example of protein pairs with a small 3DZD Euclidian distance but from different fold classes, the β class and the αβ class. Left, 3a6rA; 2.30.110.10. Right, 3h87A, 3.40.50.1010. The Euclidian distance of 3DZD was 2.4, while the TM-score was 0.254. (PDF) [file pcbi.1006969.s004.pdf]

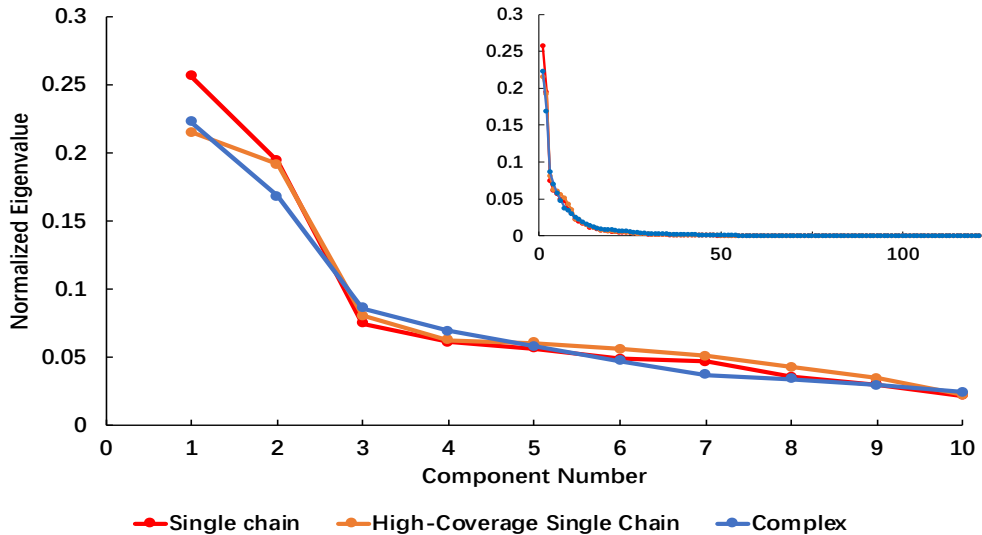

Supplement: S5 Fig — The figure shows top 10 eigenvalues of the covariance matrix sorted in the descending order. The insert shows all 121 eigenvalues. Eigenvalues of single-chain, high-coverage single-chain and complex datasets are colored in red, orange and blue, respectively. The sharp drop up to the third eigenvalue indicates that adding fourth and more eigenvalues do not add substantially more information. (PDF) [file pcbi.1006969.s005.pdf]
